# Data on corrosive water in the sources and distribution network of drinking water in north of Iran

**DOI:** 10.1016/j.dib.2017.12.057

**Published:** 2018-01-04

**Authors:** Javad Alimoradi, Dariush Naghipour, Hossein Kamani, Ghorban Asgari, Mohammad Naimi-Joubani, Seyed Davoud Ashrafi

**Affiliations:** a School of Health, Guilan University of Medical Sciences, Rasht, Iran; b Health Promotion Research Center, Zahedan University of Medical Sciences, Zahedan, Iran; c Social Determinants of Health Research Center (SDHRC), Department of Environmental Health Engineering, Hamadan University of Medical Sciences, Hamadan, Iran; d Research Center of Health and Environment, Guilan University of Medical Sciences, Rasht, Iran

**Keywords:** Drinking water, Corrosive water, Scaling potential, Amlash, Rudsar

## Abstract

This study aimed to determine the parameters of scaling and corrosion potential of drinking water in sources and distribution networks of water supply in two cities of north of Iran. The results of Amlash water sampels analysis in winter revealed that the average values of Langelier, Ryznar, Aggressive, Pockorius, and Larson- skold indices was −1.31, 9.73, 11.5, 9.74 and 0.24, respectively, but, in summer they were −1.51, 10.71, 11.36, 10.72 and 0.25, respectively. For Rudsar, the results of water sampels analysis in winter illustrated that the average values of Langelier, Ryznar, Aggressive, Pockorius, and Larson was −1.12, 9.69, 11.33, 9.19 and 0.16, respectively, while, in summer they were −1.05, 10.04, 11.92, 10.18 and 0.19, respectively. The beneficial of this data is showing the clear image of drinking water quality and can be useful for preventing the economical and safety problems relating to corrosion and scaling of drinking water.

**Specifications Table**

**Table:** 

**Subject area**	Environmental Sciences
**More specific subject area**	Drinking water chemistry
**Type of data**	Table and figure
**How data was acquired**	Measurements of all parameters was done according to standard methods based on Standard Methods for the Examination of Water and Wastewater.
	Hardness parameters, alkalinity, calcium, bicarbonate and chloride were measured by titration method.
	Digital pH meter (Metrohm) was applied for pH analyzing.
	Sulfate was measured using Hach DR5000 spectrophotometer.
	Temperature was determined by digital thermometer.
	TDS was measured by scaling method.
**Data format**	Raw, analyzed
**Experimental factors**	The data were obtained monthly in both cold and warm season, winter and summer, and the pH and temperature measured in the place other samples after taking as standard method were stored in a dark place at 4 °C temperature and transferred to laboratory under 3 hours.
**Experimental features**	All the above mentioned parameters were acquired and the levels of all indices were calculated.
**Data source location**	Guilan Province, North of Iran, Iran (Fig. 1).
**Data accessibility**	All data are available within this article.

**Value of the data**

• The data shown here can be helpful for water and wastewater companies, water resources and treatment management, and for who related with water quality engineering and management.
• The materials and ingredient of pipes, fittings and valves in distribution networks solved due to corrosive water and make some health, aesthetic and economic problems. So that, the determination of corrosion and scale potential of drinking water using reliable methods is useful for preventing of these problems.
• The zoning of the Langelier, Ryznar, Aggressive, Pockorius, and Larson indices was done to make a clear picture of the corrosion and scaling potential in the water resources and distribution network in these study area.

## 1 Data

The subject of safe drinking water is important topic in the world [1], [2], [3], [4], [5]. The data of this paper present the information about the saturation situation of water supply quality for both season of winter and summer. Five stability indices, Langelier, Ryznar, Aggressive, Pockorius, and Larson were calculated using especial equations which summarized in Table 1. In the winter for Amlash county the mean values of pH, temperature, TDS, ${HCO}_{3}^{-}$, ALK, ${SO}_{4}^{-}$, Cl^−^ and Ca^2+^ were 7.56, 11.43 °C, 156.64, 170.91, 138.38, 23.68, 17.46 and 50.69 mg/L, respectively. But, in the summer season the mean values for those parameters were 7.65, 18.18 °C, 209.97, 173.52, 141.91, 28.28, 16.71 and 34.51 mg/L, respectively (Table 2). In the other case, Rudsar county, in the winter the mean values of pH, temperature, TDS, ${HCO}_{3}^{-}$, ALK, ${SO}_{4}^{-}$, Cl^−^ and Ca^2+^ were 7.31, 11.04 °C, 248.2, 213.39, 174.34, 21.68, 13.52 and 91.97 mg/L, respectively. But, in the summer season the mean values for those parameters were 7.91, 19.46 °C, 271.04, 197.96, 162.14, 24.35, 15.23 and 68.32 mg/L, respectively (Table 3). The data reveled that in both season of winter and summer all of the water supply of Amlash were low corrosive to extremely corrosive according to Langelier, Ryznar, Pockorius, and Larson indices, but, all of the water supply except one case of sampling point in the winter, were neutral according to Aggressive index (Table 4). In the case of Rudsar, the data reveled that in both season of winter and summer all of the water supply were low corrosive to extremely corrosive according to Langelier, Ryznar, Pockorius, and Larson indices, but, all of the water supply except one case of sampling point in the winter, and six case of sampling point in the summer were neutral according to Aggressive index (Table 5). Zoning map of five calculated indices in Amlash and Rudsar were shown in Fig. 2, Fig. 3, Fig. 4, Fig. 5, Fig. 6 and Fig. 7, Fig. 8, Fig. 9, Fig. 10, Fig. 11, respectively.

**Fig. 1:**
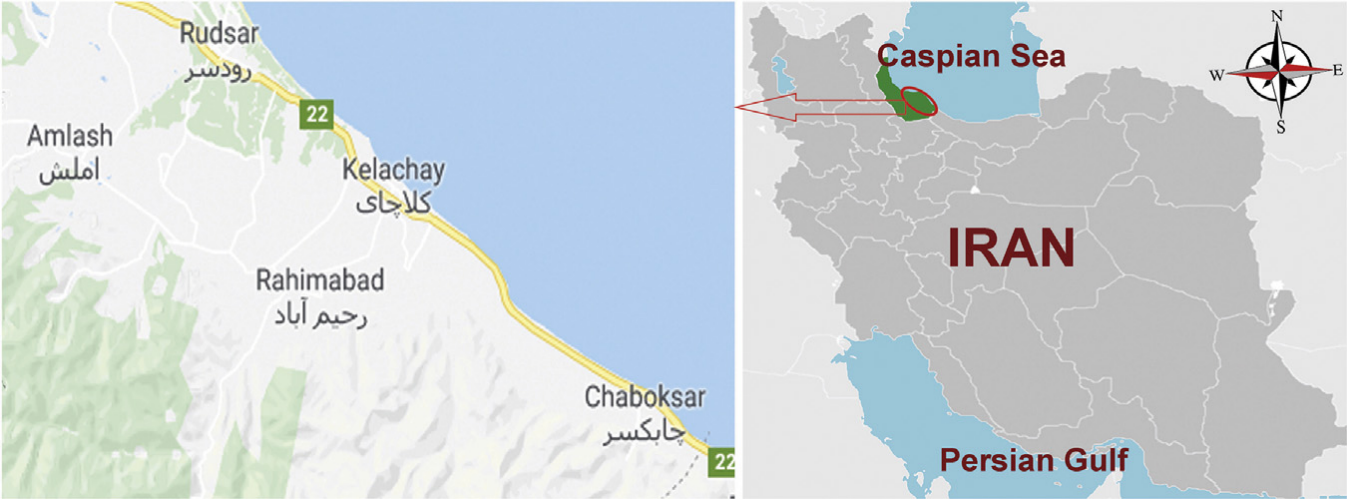
Study area; Amlash and Rudsar County, Guilan Province, north of Iran.

**Fig. 2:**
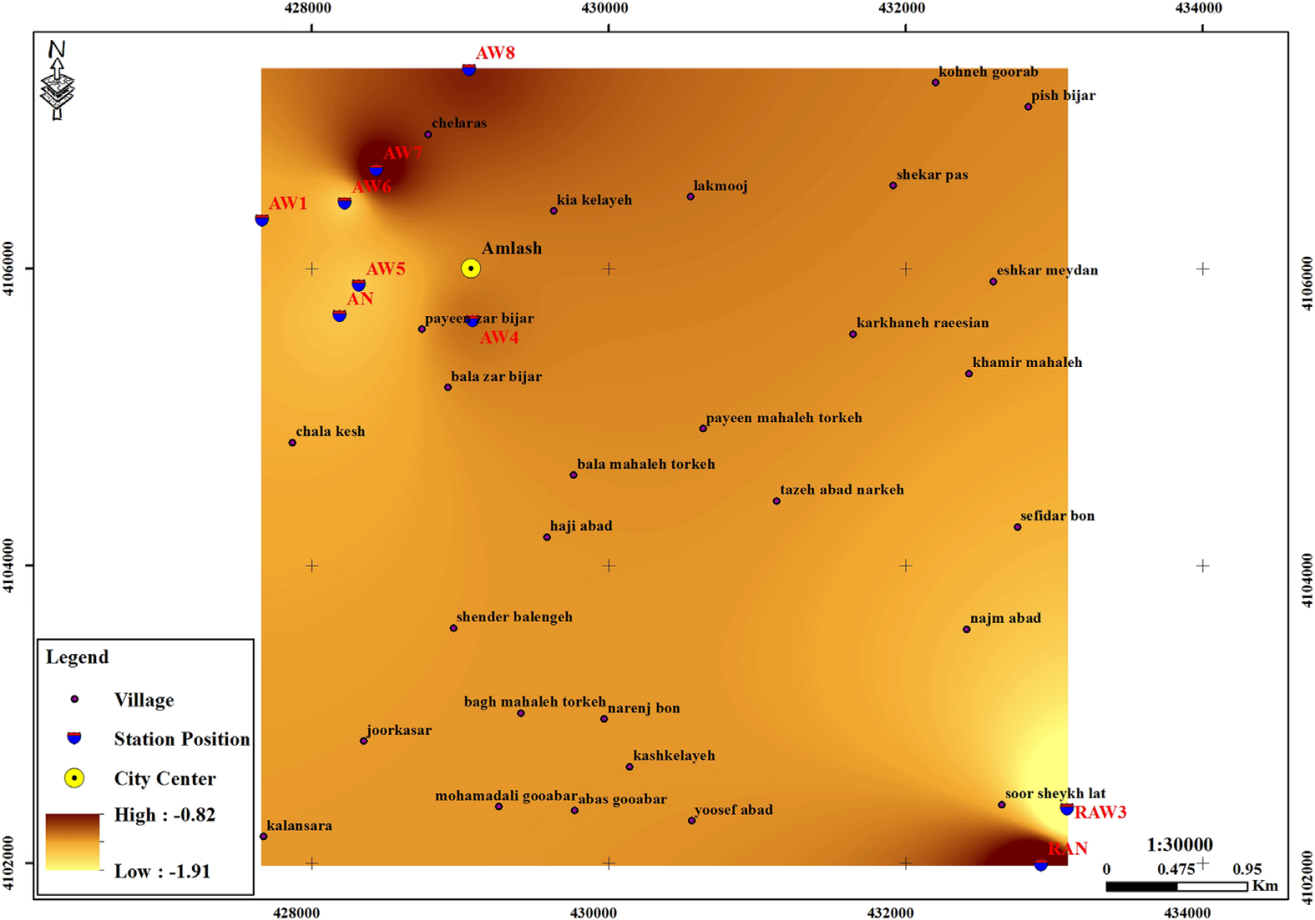
Zoning map of Langelier index in Amlash.

**Fig. 3:**
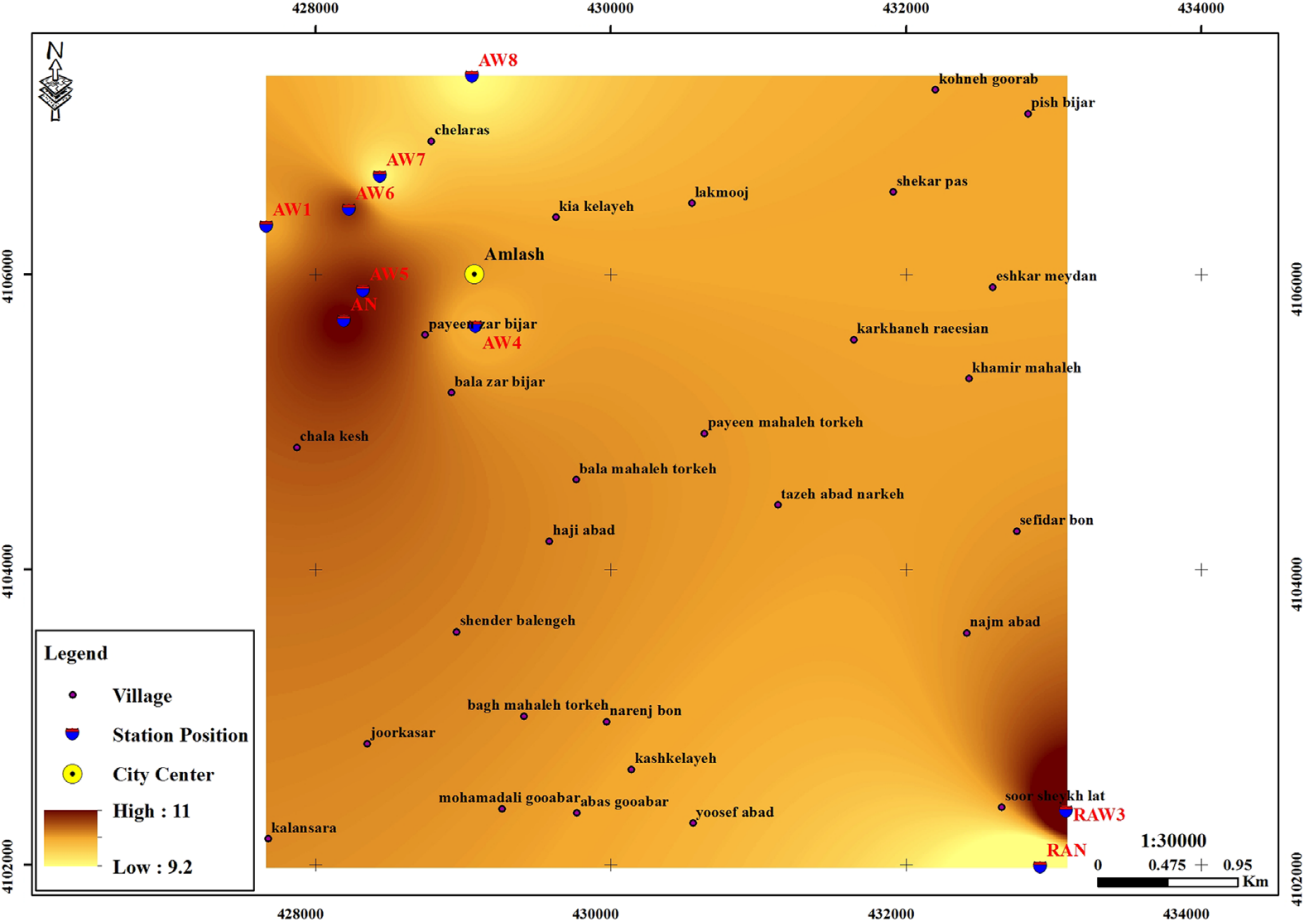
Zoning map of Ryznar index in Amlash.

**Fig. 4:**
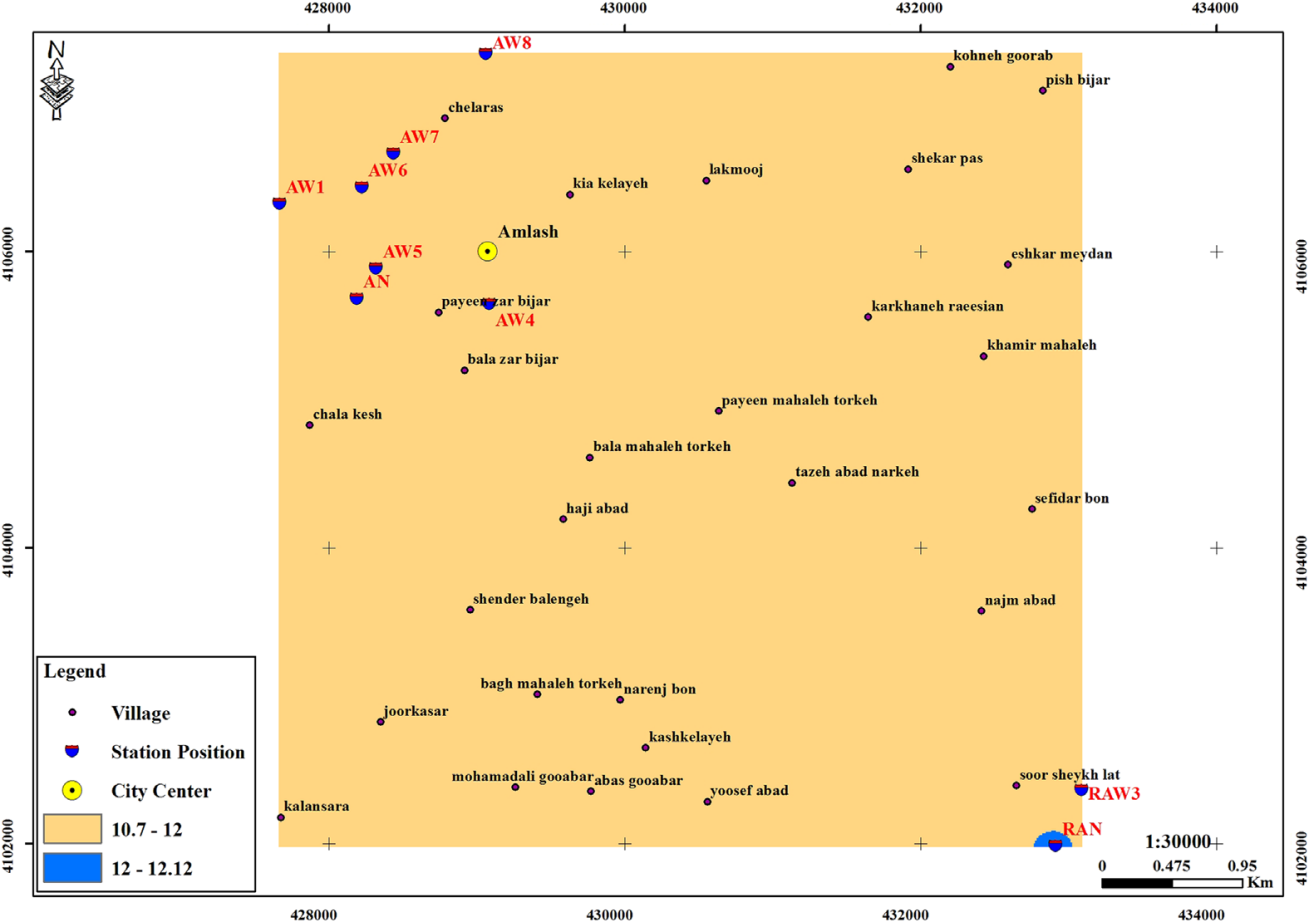
Zoning map of Aggressive index in Amlash.

**Fig. 5:**
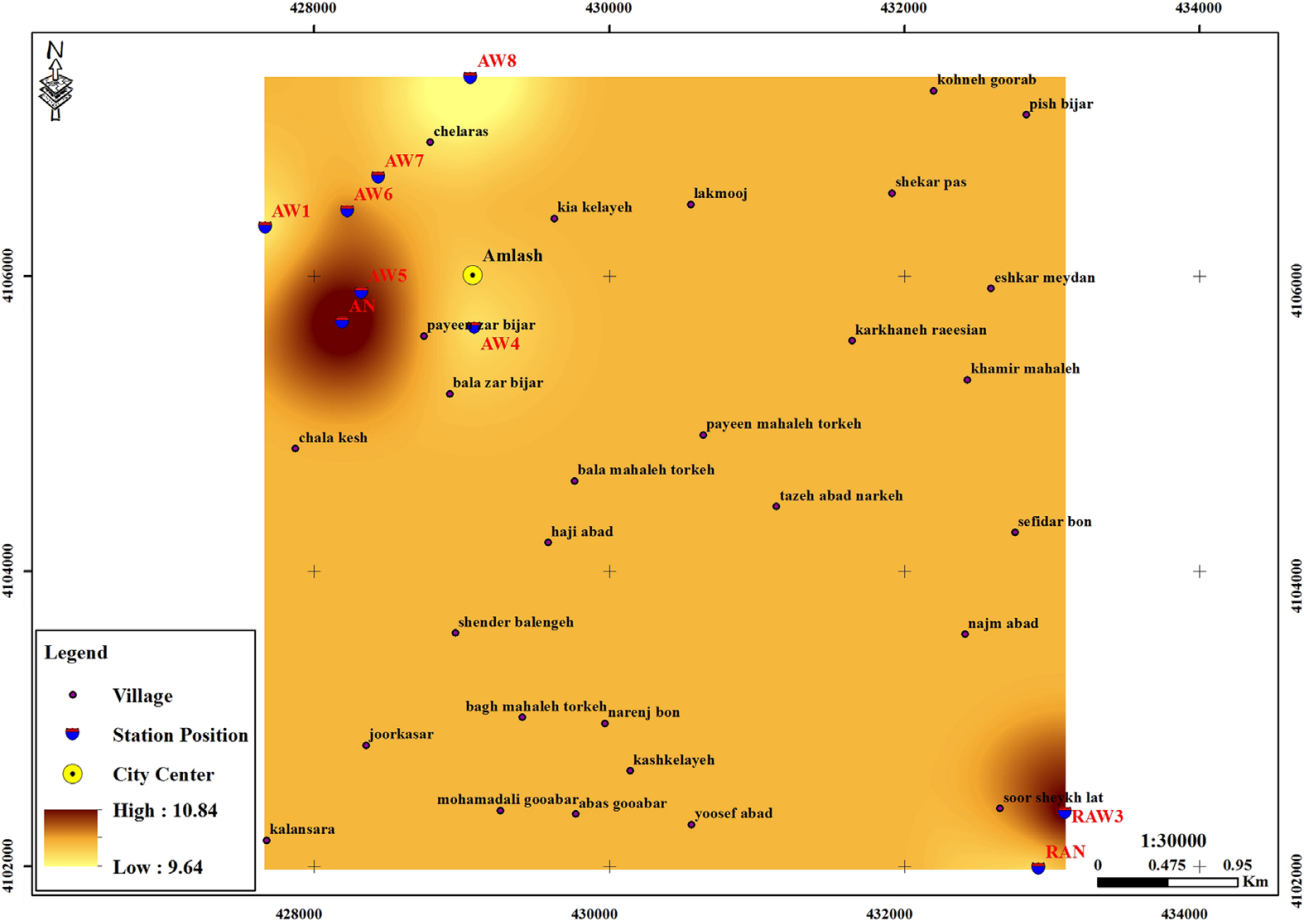
Zoning map of Pockorius index in Amlash.

**Fig. 6:**
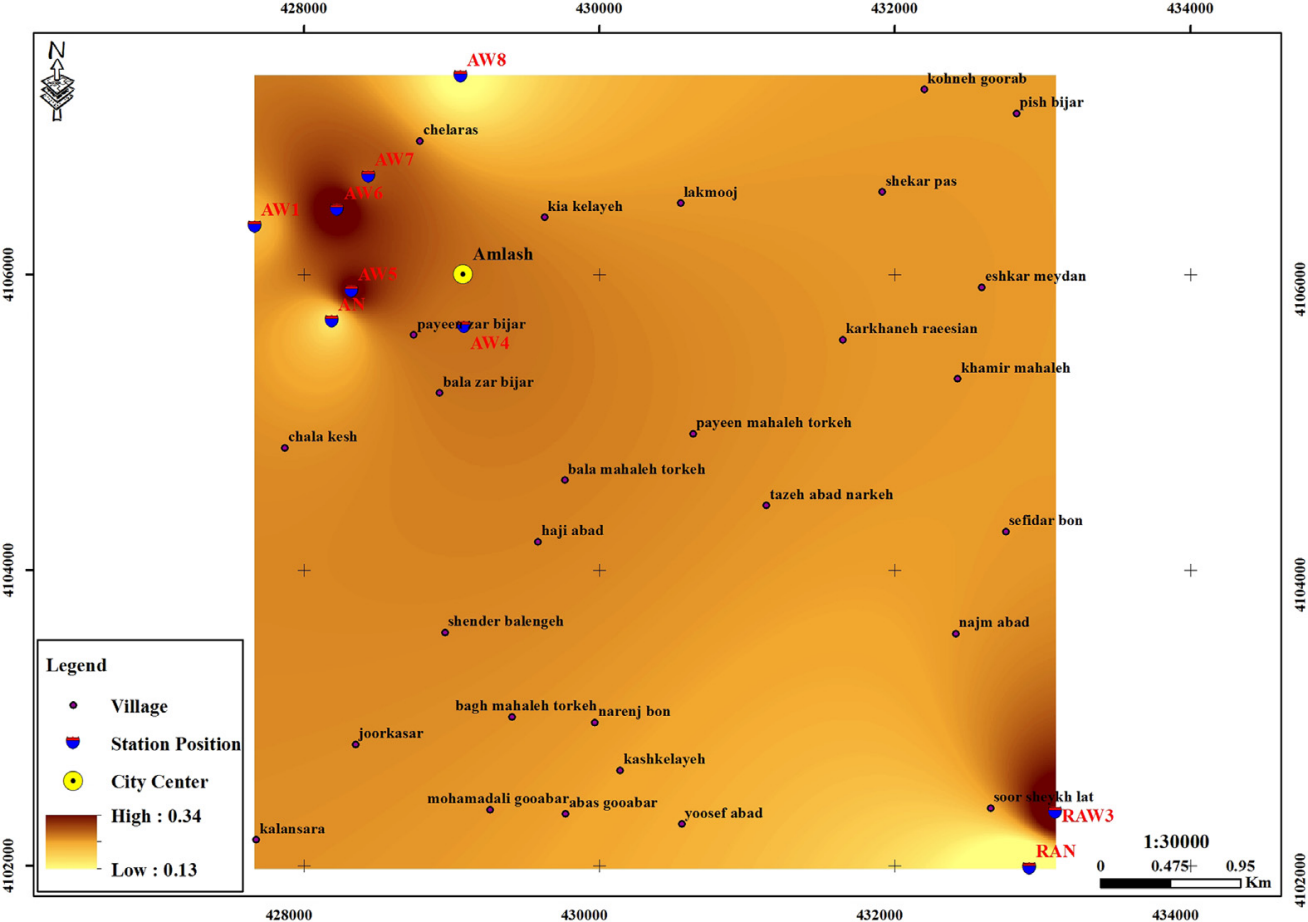
Zoning map of Larson-skold index in Amlash.

**Fig. 7:**
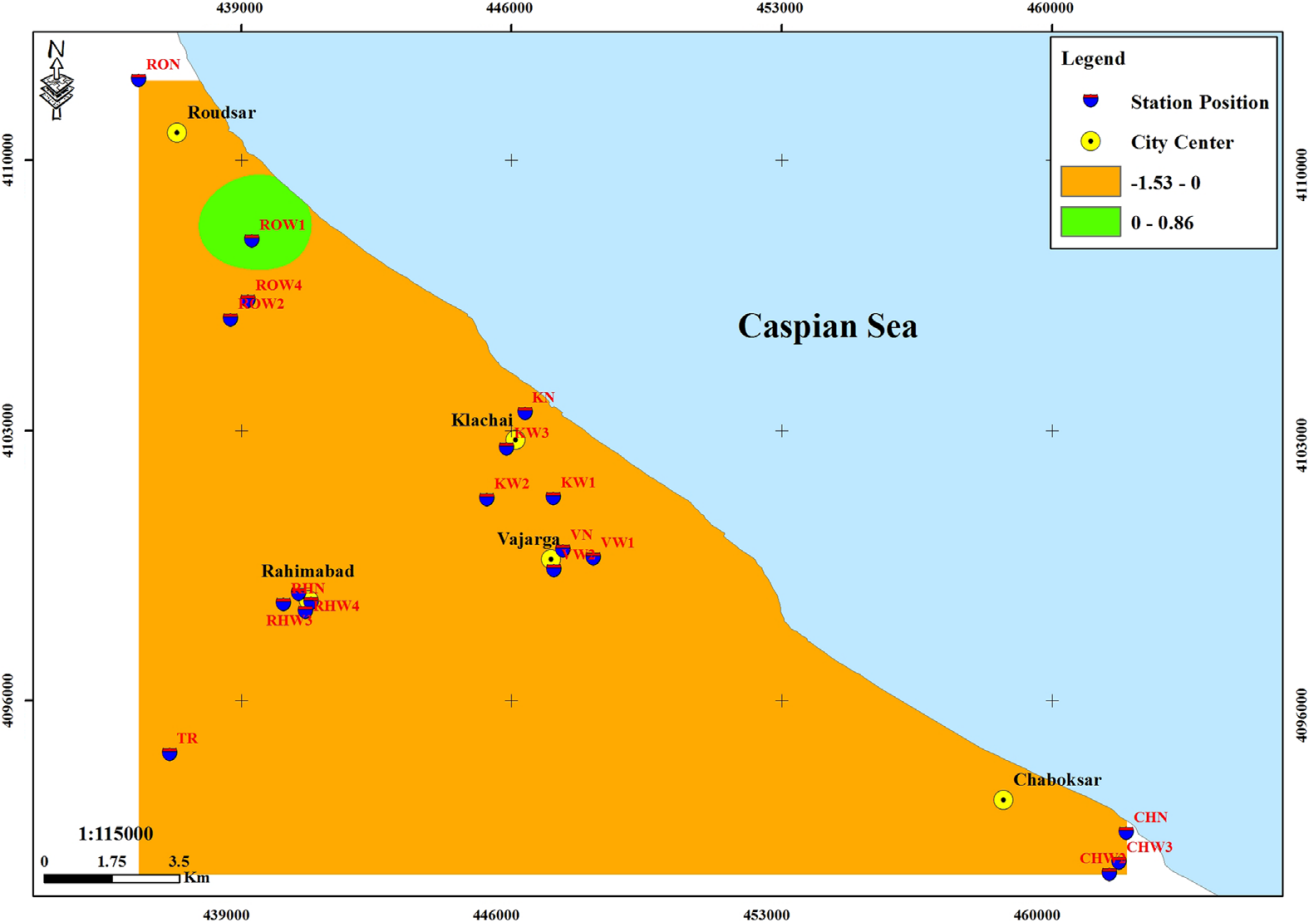
Zoning map of Langelier index in Rudsar.

**Fig. 8:**
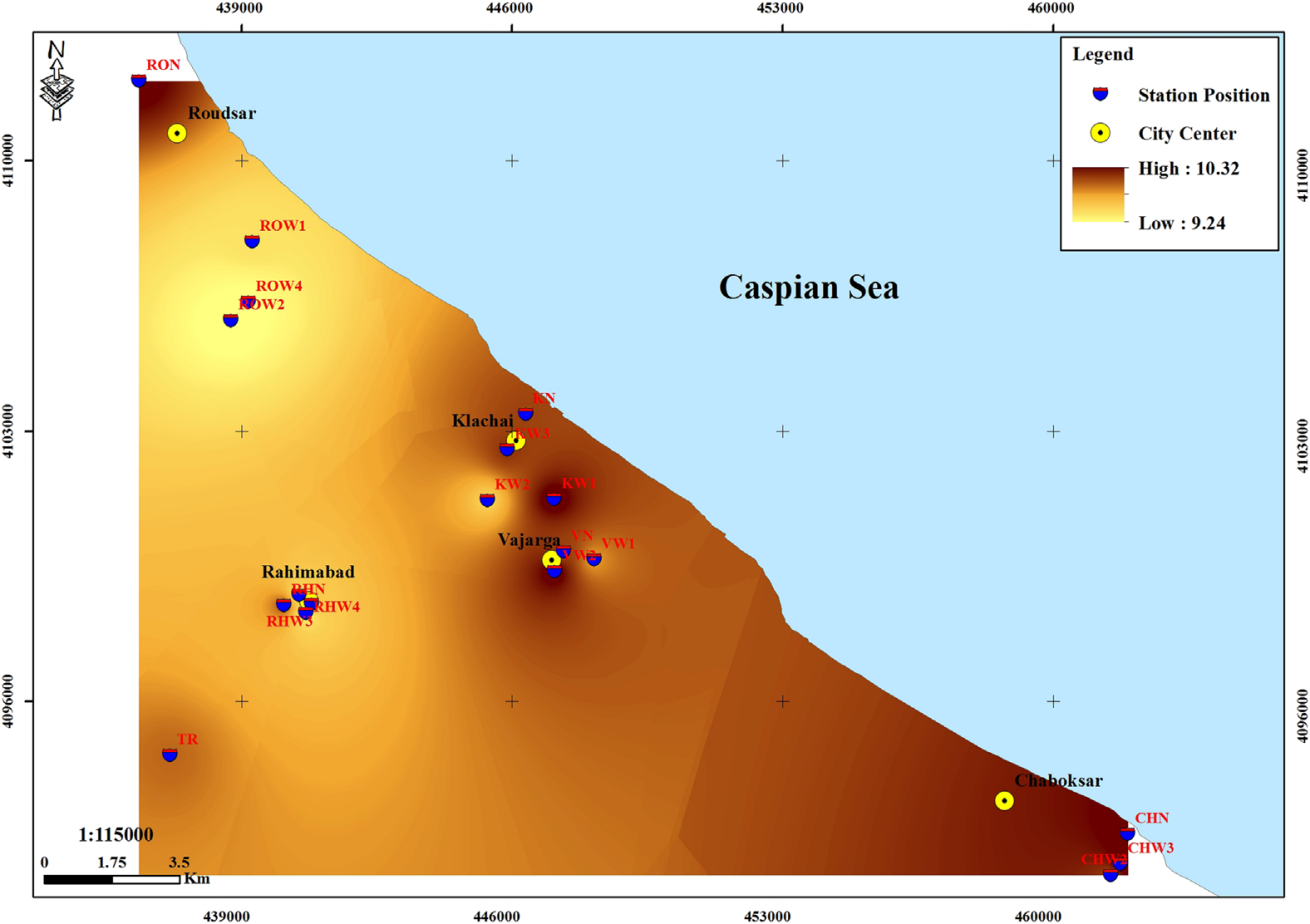
Zoning map of Ryznar index in Rudsar.

**Fig. 9:**
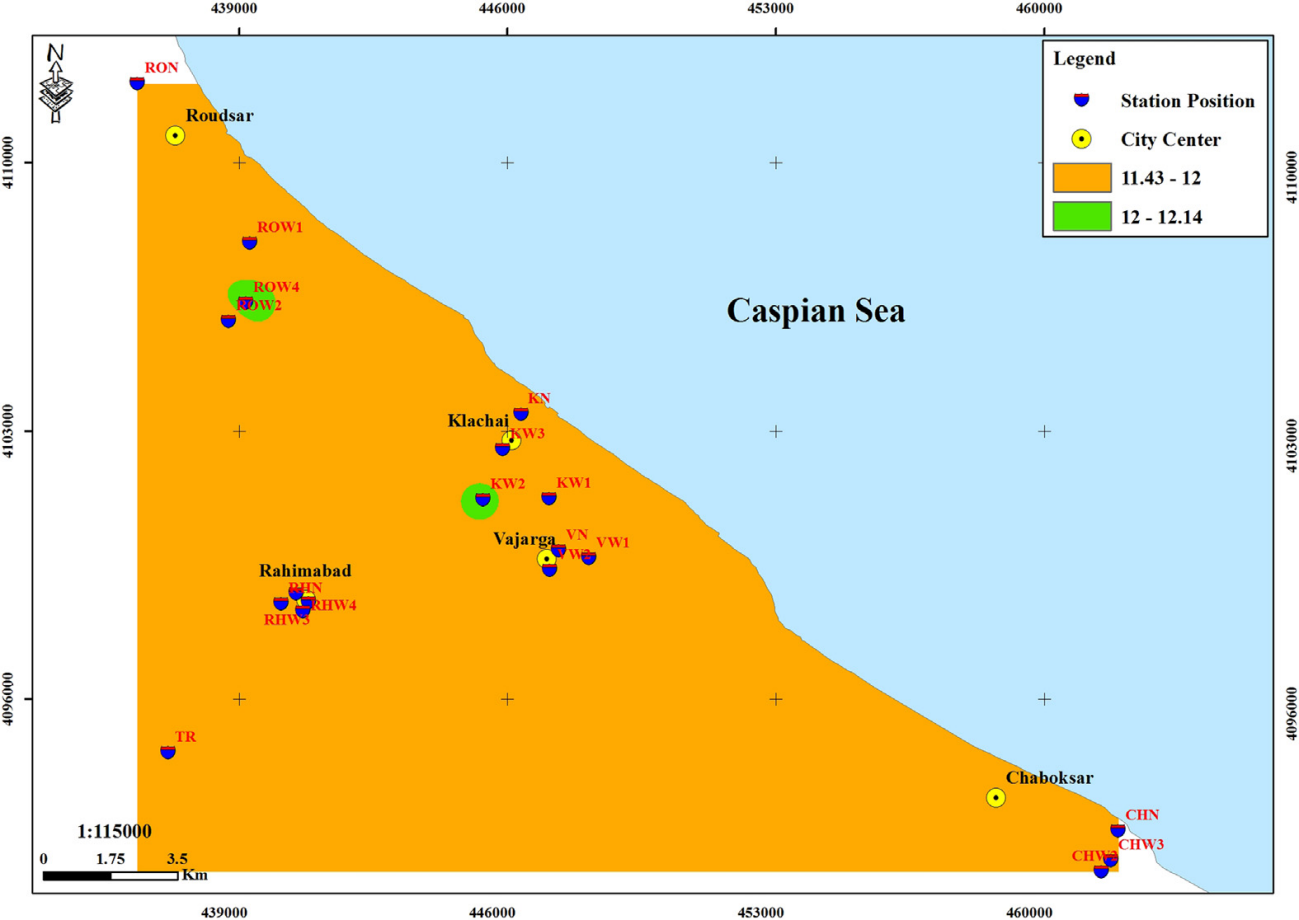
Zoning map of Aggressive index in Rudsar.

**Fig. 10:**
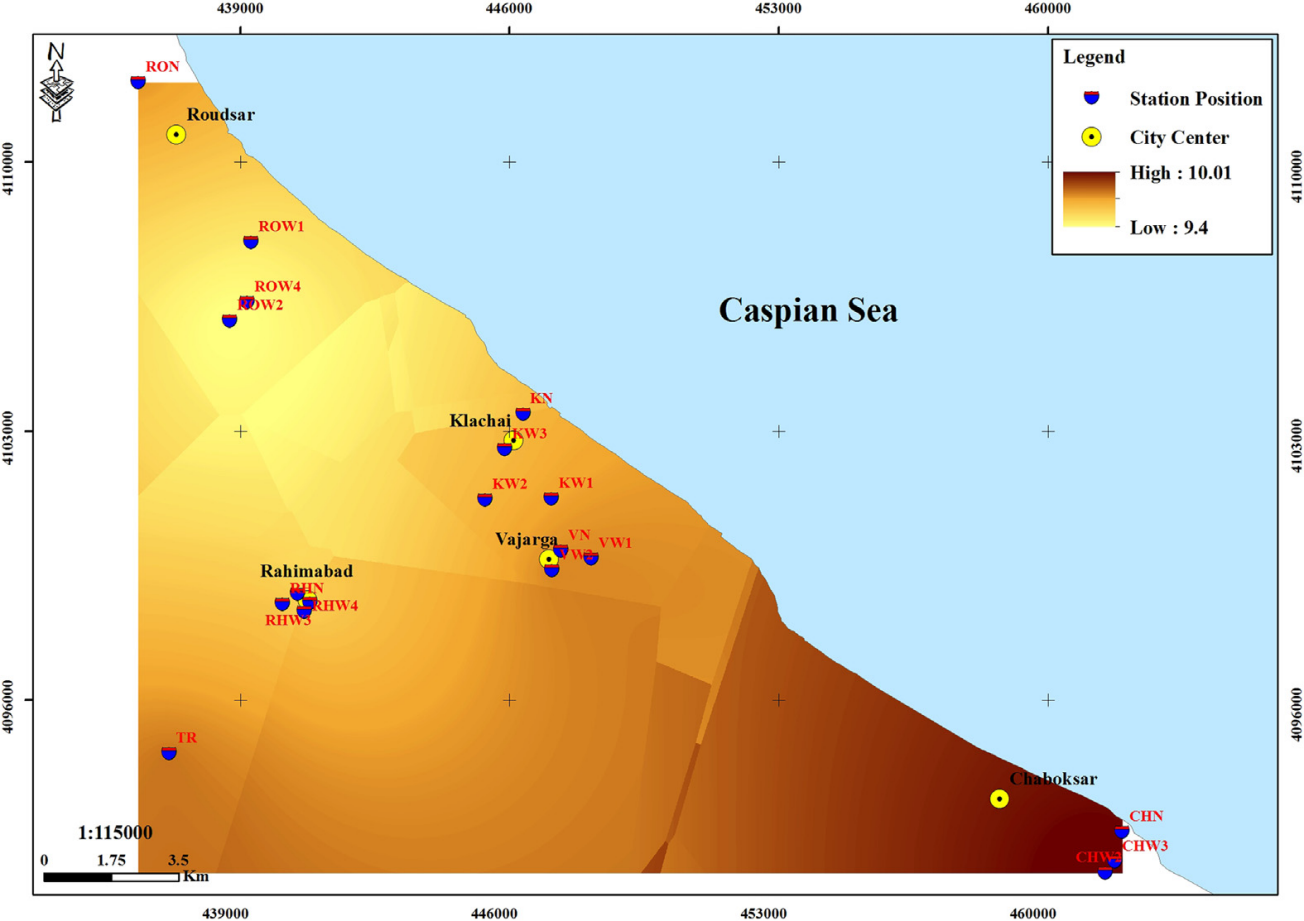
Zoning map of Pockorius index in Rudsar.

**Fig. 11:**
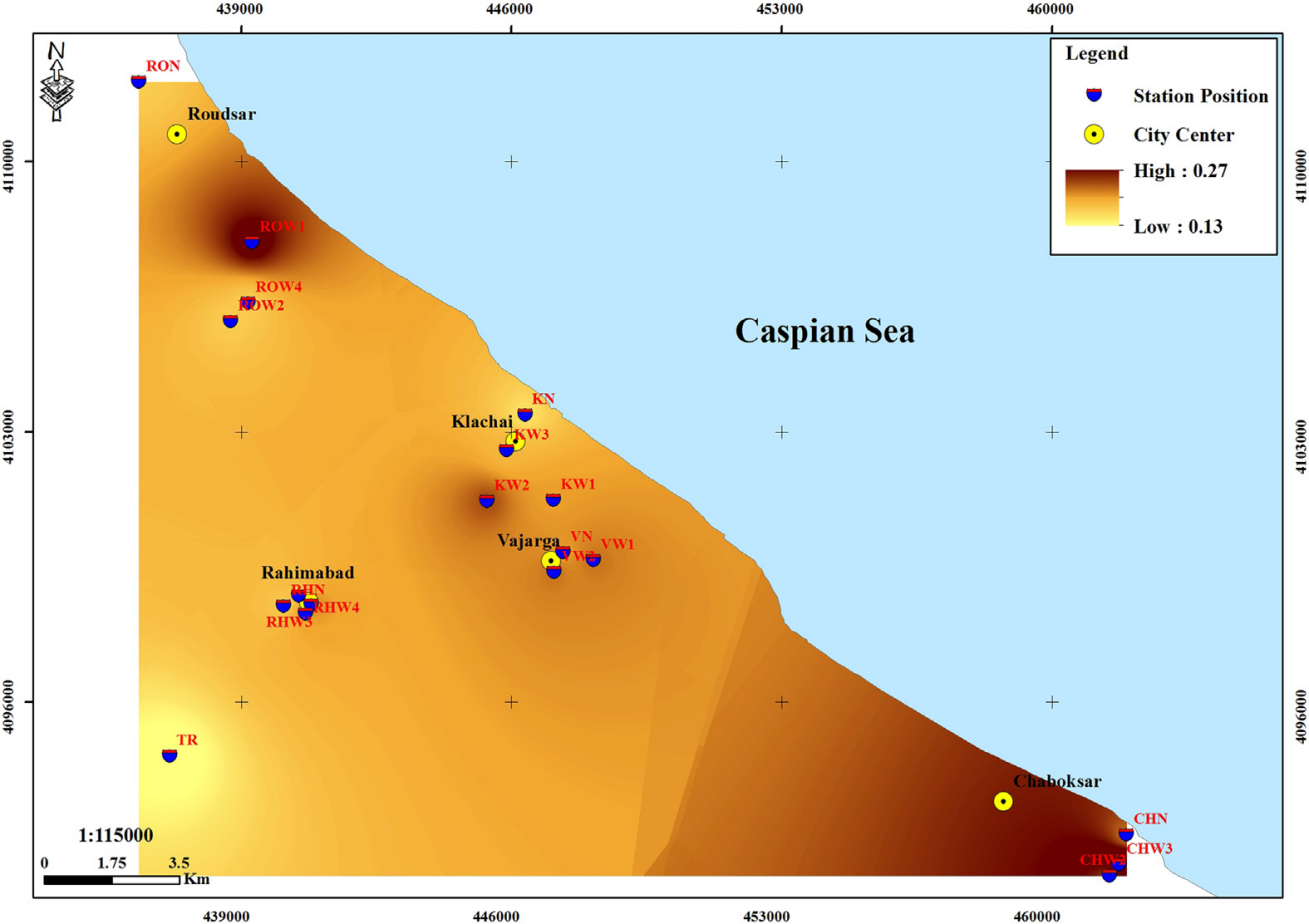
Zoning map of Larson-skold index in Rudsar.

**Table 1:** Equations and classifications of Langelier, Ryznar, Aggressive, Pockorius, and Larson-skold indices [8], [9].

**Index**	**Equation**	**Value**	**Water situation**
Langelier saturation	LSI=pH – pH_S_	LSI<0	Corrosive
	pH_S_=(9.3+A+B)−(C+D)	LSI=0	Equilibrium
	A=(log[TDS]−1)/10	LSI>0	Scaling
		
	B=−13.2(log(°C+273))+34.55		
	C= log [Ca^+2^+CaCO_3_]−0.4		
	D= log [Alkalinity as CaCO_3_]		
			
Ryznar stability	RSI=2pH_S_−pH	RSI<5.5	Heavy scale formation
		5.5< RSI<6.2	Some scale
		6.2< RSI<6.8	Non-scaling or corrosive
		6.8< RSI<8.5	Corrosive
		RSI>8.5	Extremely corrosive
			
Aggressive	AI=pH+log [(Ca^+2^) (Alk)]	AI<10	Highly corrosive
		10<AI<12	Moderate corrosive
		AI>12	Scaling
			
Puckorius scaling	PI=2pH_S_−pHeq	PSI>7	Corrosive
		
	pH_eq_=1.465 log (Alkalinity)+4.54	PSI<6	Scaling
	Alkalinity=[HCO_3_^−^]+2[CO_3_^-2^]+[OH^-^]		
			
Larson-skold	LS=[C_(CL_^-^_)_+C_(SO4_^-2^_)_] / [C_(HCO3_^-^_)_+C_(CO3_^-2^_)_]	LI>1.2	Corrosive
		
	C=Concentration (mg/L)	1.2>LI>0.8	Moderate corrosive
		LI<0.8	Low corrosive

**Table 2:** Values of analyzed parameters and calculated indices in two seasons in Amlash.

	**pH**	**Temp°C**	**TDSmg/L**	**HCO**_**3**_^**−**^**mg/L**	**ALKmg/L CaCO**_**3**_	**SO**_**4**_^**−**^**mg/L**	**Cl**^**−**^**mg/L**	**Ca**^**2+**^**mg/L**	**LSI**	**RSI**	**AI**	**PSI**	**LI**
**Winter**													
AW1	7.38	9.69	175.4	216.54	176.44	25.47	18.13	61.69	0.14	9.6	11.42	9.15	−1.1
RAW3	7.01	13.53	113.54	109.81	79.49	25.34	15.06	35.92	0.36	10.94	10.52	10.55	−1.96
AW4	7.42	14.74	205.03	231.24	188.99	14.64	43.06	56.8	0.25	9.93	11.45	9.48	−1.25
AW5	7.51	13.67	186.28	121.06	98.48	27.54	15.86	54.51	0.36	10.45	11.23	10.51	−1.46
AW6	7.57	10.29	182.56	165.54	134.22	35.67	18.25	44.32	0.32	10.02	11.34	9.94	−1.22
AW7	8.18	8.31	114.06	141.05	115.36	27.5	15.57	53.21	0.3	8.98	11.97	9.61	−0.39
AW8	7.61	8.9	165.54	212.89	172.44	21.04	13.04	62.86	0.16	9.24	11.65	9.04	−2.44
AN	7.52	14.39	161.1	147.99	123.7	20.77	9.63	43.68	0.2	10.34	11.25	10.26	−1.41
RAN	7.9	9.42	106.31	192.12	156.38	15.15	8.57	43.26	0.12	8.09	12.67	9.18	−0.57
Min	7.01	8.31	106.31	109.81	79.49	14.64	8.57	35.92	0.12	8.09	10.52	9.04	−2.44
Max	8.18	14.74	205.03	231.24	188.99	35.67	43.06	62.86	0.36	10.94	12.67	10.55	−0.39
Mean	7.56	11.43	156.64	170.91	138.38	23.68	17.46	50.69	0.24	9.73	11.5	9.74	−1.31
St.Dev.	0.32	2.58	36.3	44.15	37.61	6.6	10.17	9.29	0.09	0.86	0.58	0.59	0.63
**Summer**													
AW1	7.55	18.38	237.48	180.48	147.69	34.29	15.73	37.08	0.27	10.84	11.28	10.68	−1.64
RAW3	7.32	17.98	168.62	136.75	111.66	28.56	14.88	33.8	0.31	11.07	10.89	10.85	−1.87
AW4	7.56	18.14	256.09	205.87	168.38	29.61	31.46	38.18	0.29	10.43	11.67	10.49	−1.28
AW5	7.72	18.3	260.21	199.8	164	32.38	20.78	37.04	0.28	10.7	11.47	10.66	−1.49
AW6	7.45	18.28	227.27	169.09	137.5	39.03	19.44	37.51	0.34	10.96	11.14	10.74	−1.75
AW7	7.62	18.24	269.73	188.19	154.16	30.85	16.77	40.99	0.25	10.74	11.41	10.62	−1.56
AW8	7.54	18.76	193.27	189.93	154.99	28.82	12.56	47.1	0.21	10.43	11.4	10.23	−1.44
AN	7.95	17.19	150.18	124.95	102.1	17.26	9.43	18.71	0.22	10.94	11.2	11.43	−1.49
RAN	8.15	18.4	126.96	166.7	136.72	13.79	9.36	20.19	0.14	10.31	11.85	10.8	−1.08
Min	7.32	17.19	126.96	124.95	102.1	13.79	9.36	18.71	0.14	10.31	10.89	10.23	−1.87
Max	8.15	18.76	269.73	205.87	168.38	39.03	31.46	47.1	0.34	11.07	11.85	11.43	−1.08
Mean	7.65	18.18	209.97	173.52	141.91	28.28	16.71	34.51	0.25	10.71	11.36	10.72	−1.51
St.Dev.	0.25	0.42	52.12	27.49	22.6	7.97	6.8	9.29	0.06	0.26	0.28	0.32	0.23

**Table 3:** Values of analyzed parameters and calculated indices in two seasons in Rudsar.

	**pH**	**Temp °C**	**TDS mg/L**	**HCO**_**3**_^**−**^**mg/L**	**ALK mg/L CaCO**_**3**_	**SO**_**4**_^**−**^**mg/L**	**Cl**^**−**^**mg/L**	**Ca**^**2+**^**mg/L**	**LSI**	**RSI**	**AI**	**PSI**	**LI**
**Winter**													
RON	7.18	11.53	243.25	186.18	152.39	20.94	10.53	76.73	0.17	10.16	10.16	9.61	−1.48
KN	7.12	12.42	327.87	224.49	184.1	18.84	8.17	77.19	0.12	10.06	11.43	9.32	−1.47
RHN	7.75	11.77	133.5	173.45	141.21	20.38	11.08	46.08	0.18	9.61	11.56	9.67	−0.92
VN	7.25	11.89	335.59	244.95	199.55	20.43	18.47	116.62	0.15	9.8	11.61	9.14	−0.13
CHN	6.89	12.26	323.43	224.55	183.94	25.23	13.65	110.41	0.17	10.28	11.2	9.32	−1.69
TR	7.81	10.17	137.32	183.49	149.83	18.77	5.92	49.82	0.13	9.3	9.39	9.39	−0.74
ROW1	7.53	8.45	195.81	212.12	173.1	28.64	15.17	96.54	0.2	9.05	11.75	8.76	−0.76
ROW2	7.23	7.93	164.35	230.78	188.61	19.76	13.44	102.6	0.14	8.99	11.51	8.35	−0.88
ROW4	7.7	11.06	207.93	215.28	176.27	22.13	9.59	105.19	0.15	9.08	11.96	8.94	−0.69
KW1	6.65	11.17	317.87	223.67	182.88	28/5	11.23	98.19	0.17	10.53	10.9	9.33	−1.93
KW2	7.11	11.03	307.01	241.36	196.88	24.72	12.05	107.57	0.15	9.85	11.44	9.07	−1.36
KW3	6.73	11.19	292.88	258.04	210.83	20.47	14.04	107.13	0.13	10.18	11.08	8.97	−1.72
RHW2	7.48	12.85	170.66	227.89	186.21	17.8	17.76	94.14	0.14	9.3	11.72	8.92	−0.91
RHW3	7.1	13.28	158.42	223.68	182.99	21.19	12.87	99.58	0.15	9.61	11.36	8.87	−1.25
RHW4	7.34	8.23	178.16	220.6	179.88	21.67	12.45	96.06	0.15	9.09	11.57	8.59	−0.87
VW1	7.69	9.81	313.91	204.97	167.49	17.29	21.58	101.37	0.19	9.41	11.91	9.3	−0.86
VW2	7.27	10.53	292.26	210.18	171.5	23.87	15.23	86.86	0.18	9.93	11.42	9.4	−1.33
CHW2	7.52	11.16	301.17	194.24	158.99	22.48	15.42	93.84	0.19	9.78	11.68	9.53	−1.13
CHW3	7.67	13.2	314.47	154.63	125.83	25.77	18.23	81.61	0.28	10.13	11.68	10.19	−1.23
Min	6.65	7.93	133.5	154.63	125.83	17.29	5.92	46.08	0.12	8.99	9.39	8.35	−1.93
Max	7.81	13.28	335.59	258.04	210.83	28.64	21.58	116.62	0.28	10.53	11.96	10.19	−0.13
Mean	7.31	11.04	248.2	213.39	174.34	21.68	13.52	91.97	0.16	9.69	11.33	9.19	−1.12
St.Dev.	0.34	1.57	74.25	25.74	21.1	3.3	3.83	18.86	0.35	0.46	0.62	0.42	0.43
**Summer**													
RON	7.81	20.15	236.07	173.42	142.39	20.15	9.08	51.31	0.16	10.4	11.67	10.52	−1.29
KN	7.92	20.86	338.86	208.23	170.83	24.08	12.58	85.04	0.18	10.04	12.08	10.15	−1.06
RHN	8.03	18.32	139.93	163.45	134.16	16.96	5.67	22.55	0.13	10.43	11.5	10.8	−1.2
VN	7.78	18.41	351.11	202.19	165.77	24.11	18.17	70.94	0.21	10.3	11.84	10.29	−1.25
CHN	7.62	17.99	313.3	201.2	162.66	24.73	17.87	77.05	0.21	10.27	11.71	10.11	−1.32
TR	7.78	17.62	140.96	166.65	136.61	15.11	7.91	23.46	0.14	10.58	11.29	10.7	−1.39
ROW1	7.92	20.02	244.79	200.76	163.33	28.79	22.1	75.14	0.25	9.87	12	10.01	−0.97
ROW2	8.07	19.98	221.34	213.25	173.88	26.15	12.22	80.99	0.19	9.5	12.22	9.75	−0.71
ROW4	8.07	19.89	235.14	207.42	169.44	24.38	12.79	85.1	0.18	9.52	12.23	9.8	−0.72
KW1	7.83	19.3	315.08	202.13	170.27	26.34	12.3	78.54	0.19	10.07	11.95	10.09	−1.12
KW2	8.7	20.09	330.82	192.8	157.61	29.62	16.72	88.33	0.24	9.24	12.84	10.19	−0.26
KW3	7.82	20.05	315.33	214.79	175.77	26.35	18.54	86.2	0.21	10	11.99	9.99	−1.09
RHW2	7.93	19.16	211.86	210.06	171.66	24.44	14.44	80.55	0.18	9.58	12.07	9.7	−0.82
RHW3	7.76	19.25	188.02	206.59	168.99	26.17	21.51	76.71	0.23	9.71	11.87	9.67	−0.97
RHW4	7.95	20.36	198.98	216	176.72	24.12	19.93	73.83	0.2	9.61	12.06	9.73	−0.82
VW1	7.8	19.22	350.7	215.61	175.88	23.84	20.45	71.92	0.2	10.21	11.9	10.19	−1.2
VW2	7.84	20.25	362.7	204.62	167.83	23.69	16.54	47.26	0.19	10.67	11.73	10.72	−1.41
CHW2	7.96	19.43	328.07	195.73	160.83	26.67	14.26	58.79	0.21	10.3	11.93	10.48	−1.16
CHW3	7.78	19.44	326.76	166.41	136.11	27.01	16.34	64.48	0.26	10.52	11.72	10.64	−1.36
Min	7.62	17.62	139.93	163.45	134.16	15.11	5.67	22.55	0.13	9.24	11.29	9.67	−1.41
Max	8.7	20.86	362.7	216	176.72	29.62	22.1	88.33	0.26	10.67	12.84	10.8	−0.26
Mean	7.91	19.46	271.04	197.96	162.14	24.35	15.23	68.32	0.19	10.04	11.92	10.18	−1.05
St.Dev.	0.22	0.86	73.31	17.42	14.19	3.59	4.61	19.55	0.03	0.41	0.32	0.37	0.29

**Table 4:** The condition of drinking water in view of scaling and corrosion indices in Amlash.

**Sampling point**	**LSI**	**RSI**	**AI**	**PSI**	**LI**
**Winter**					
AW1	Corrosive	Extremely corrosive	Moderate corrosive	Corrosive	Corrosive
RAW3	Corrosive	Extremely corrosive	Moderate corrosive	Corrosive	Corrosive
AW4	Corrosive	Extremely corrosive	Moderate corrosive	Corrosive	Corrosive
AW5	Corrosive	Extremely corrosive	Moderate corrosive	Corrosive	Corrosive
AW6	Corrosive	Extremely corrosive	Moderate corrosive	Corrosive	Corrosive
AW7	Corrosive	Extremely corrosive	Moderate corrosive	Corrosive	Corrosive
AW8	Corrosive	Extremely corrosive	Moderate corrosive	Corrosive	Corrosive
AN	Corrosive	Extremely corrosive	Moderate corrosive	Corrosive	Corrosive
RAN	Corrosive	Extremely corrosive	Scaling	Corrosive	Corrosive
**Summer**					
AW1	Corrosive	Extremely corrosive	Moderate corrosive	Corrosive	Corrosive
RAW3	Corrosive	Extremely corrosive	Moderate corrosive	Corrosive	Corrosive
AW4	Corrosive	Extremely corrosive	Moderate corrosive	Corrosive	Corrosive
AW5	Corrosive	Extremely corrosive	Moderate corrosive	Corrosive	Corrosive
AW6	Corrosive	Extremely corrosive	Moderate corrosive	Corrosive	Corrosive
AW7	Corrosive	Extremely corrosive	Moderate corrosive	Corrosive	Corrosive
AW8	Corrosive	Extremely corrosive	Moderate corrosive	Corrosive	Corrosive
AN	Corrosive	Extremely corrosive	Moderate corrosive	Corrosive	Corrosive
RAN	Corrosive	Extremely corrosive	Moderate corrosive	Corrosive	Corrosive

**Table 5:** The condition of drinking water in view of scaling and corrosion indices in Rudsar.

**Sampling point**	**LSI**	**RSI**	**AI**	**PSI**	**LI**
**Winter**					
RON	Corrosive	Extremely corrosive	Moderate corrosive	Corrosive	Corrosive
N	Corrosive	Extremely corrosive	Moderate corrosive	Corrosive	Corrosive
RHN	Corrosive	Extremely corrosive	Moderate corrosive	Corrosive	Corrosive
VN	Corrosive	Extremely corrosive	Moderate corrosive	Corrosive	Corrosive
CHN	Corrosive	Extremely corrosive	Moderate corrosive	Corrosive	Corrosive
TR	Corrosive	Extremely corrosive	Highly corrosive	Corrosive	Corrosive
ROW1	Corrosive	Extremely corrosive	Moderate corrosive	Corrosive	Corrosive
ROW2	Corrosive	Extremely corrosive	Moderate corrosive	Corrosive	Corrosive
ROW4	Corrosive	Extremely corrosive	Moderate corrosive	Corrosive	Corrosive
KW1	Corrosive	Extremely corrosive	Moderate corrosive	Corrosive	Corrosive
KW2	Corrosive	Extremely corrosive	Moderate corrosive	Corrosive	Corrosive
KW3	Corrosive	Extremely corrosive	Moderate corrosive	Corrosive	Corrosive
RHW2	Corrosive	Extremely corrosive	Moderate corrosive	Corrosive	Corrosive
RHW3	Corrosive	Extremely corrosive	Moderate corrosive	Corrosive	Corrosive
RHW4	Corrosive	Extremely corrosive	Moderate corrosive	Corrosive	Corrosive
VW1	Corrosive	Extremely corrosive	Moderate corrosive	Corrosive	Corrosive
VW2	Corrosive	Extremely corrosive	Moderate corrosive	Corrosive	Corrosive
CHW2	Corrosive	Extremely corrosive	Moderate corrosive	Corrosive	Corrosive
CHW3	Corrosive	Extremely corrosive	Moderate corrosive	Corrosive	Corrosive
**Summer**					
RON	Corrosive	Extremely corrosive	Moderate corrosive	Corrosive	Corrosive
KN	Corrosive	Extremely corrosive	Scaling	Corrosive	Corrosive
RHN	Corrosive	Extremely corrosive	Moderate corrosive	Corrosive	Corrosive
VN	Corrosive	Extremely corrosive	Moderate corrosive	Corrosive	Corrosive
CHN	Corrosive	Extremely corrosive	Moderate corrosive	Corrosive	Corrosive
TR	Corrosive	Extremely corrosive	Moderate corrosive	Corrosive	Corrosive
ROW1	Corrosive	Extremely corrosive	Moderate corrosive	Corrosive	Corrosive
ROW2	Corrosive	Extremely corrosive	Scaling	Corrosive	Corrosive
ROW4	Corrosive	Extremely corrosive	Scaling	Corrosive	Corrosive
KW1	Corrosive	Extremely corrosive	Moderate corrosive	Corrosive	Corrosive
KW2	Corrosive	Extremely corrosive	Scaling	Corrosive	Corrosive
KW3	Corrosive	Extremely corrosive	Moderate corrosive	Corrosive	Corrosive
RHW2	Corrosive	Extremely corrosive	Scaling	Corrosive	Corrosive
RHW3	Corrosive	Extremely corrosive	Moderate corrosive	Corrosive	Corrosive
RHW4	Corrosive	Extremely corrosive	Scaling	Corrosive	Corrosive
VW1	Corrosive	Extremely corrosive	Moderate corrosive	Corrosive	Corrosive
VW2	Corrosive	Extremely corrosive	Moderate corrosive	Corrosive	Corrosive
CHW2	Corrosive	Extremely corrosive	Moderate corrosive	Corrosive	Corrosive
CHW3	Corrosive	Extremely corrosive	Moderate corrosive	Corrosive	Corrosive

## 2 Experimental design, materials and methods

### 2.1 Study area description

The selected study area were Amlash (Population; 18,580) and Rudsar (Population; 93,970) county, located in Guilan, the major province in north of Iran, which shown in Fig. 1 [6]. In the both county of Amlash and Rudsar, the climate is warm and temperate and in winter, there is much more rainfall than in summer. The average annual rainfall in Amlash and Rudsar is 1162 and 1178 mm, respectively. In addition, the average annual temperature in both county is 15.8 °C. Most of the water distribution network in Amlash and Rudsar are made of metal materials with the length of 97 and 400 km, respectively.

### 2.2 Sample collection and analytical procedures

This research was a cross-sectional study during two season of winter and summer in 2017, and each month one sample were taken from each sample point. Therefore, fifty two samples (27 in winter and 27 in summer) were taken from nine sample point of Amlash, and one hundred and fifteen samples (57 in winter and 57 in summer) were taken from nineteen sample point of Rudsar. All measurements of the above parameters were carried out according to standard methods manual [7]. The samples were obtained monthly in winter and summer, and the pH and temperature were measured in the sampling place, other samples were stored in a dark cold box (4 °C) and transferred to laboratory of school of health under 3 h. Hardness parameters, alkalinity, calcium, bicarbonate and chloride were measured by titration method according to Standard Methods for the Examination of Water and Wastewater. Sulfate was measured using spectrophotometry method and total dissolved solid was measured by scaling method. Statistical analysis of the data was done using Microsoft Excel 2013 and spatial distribution of five calculated indices were done using Arc GIS.
